# ACCESS TO PREFERRED POSTACUTE CARE PATHWAYS FOR PATIENTS WITH ALZHEIMER’S DISEASE AND RELATED DEMENTIAS

**DOI:** 10.1093/geroni/igad104.0012

**Published:** 2023-12-21

**Authors:** Taylor Bucy, John McHugh, Dori Cross

**Affiliations:** University of Minnesota School of Public Health, Minneapolis, Minnesota, United States; Mailman School of Public Health, Columbia University, New York City, New York, United States; University of Minnesota School of Public Health, Minneapolis, Minnesota, United States

## Abstract

Hospital-to-skilled nursing facility (SNF) transitions are fraught with coordination challenges, motivating selective investments by hospitals to improve transitional care practices with “preferred” (e.g. high-volume) SNF partners. Because these partners have some agency in which patients they admit, it is unclear whether Medicare beneficiaries with social or clinical complexities, such as individuals with Alzheimer’s disease and related dementias (ADRD) have equitable access to these preferred SNFs. To answer this question, we use a linear probability choice model to test the differential effect of a SNF’s “preferredness” on patient placement for ADRD vs non-ADRD beneficiaries. We use a 1:1 matched sample of ADRD and non-ADRD fee-for-service beneficiaries (N=76,762) to account for other primary factors affecting placement (i.e. discharging hospital, beneficiaries’ home address). After controlling for SNF characteristics, the estimated effect of a SNF being “preferred” on likelihood of placement was 12.7% lower for patients with ADRD (0.092 vs 0.103 for non-ADRD; p< 0.001). Simulation results suggest that, as a preferred SNF is assumed to have an increasing percentage of a hospital’s discharges, the likelihood of a patient being discharged there grows much faster for non-ADRD patients compared to those with ADRD. Our findings show that ADRD patients may have unequal access to SNFs that are receiving the most investments from hospitals in the form of transitional care improvements. Policymakers should consider systemic investments that benefit all SNF partners, not just the most preferred based on shared discharges, while also weighing the costs of dispersing these investments too broadly.

